# CD19 expression is preserved following CD19-directed monoclonal antibody therapy with tafasitamab

**DOI:** 10.3389/fimmu.2026.1878229

**Published:** 2026-09-03

**Authors:** Johannes Duell, Diana Alvarez Arias, Wolfram Klapper, Sarah Reinke, Florian Eisele, Thomas Stauffer Larsen, Max S. Topp, Beth Rumberger, TaeHyung Kim, Andreas Rosenwald, Hilka Rauert-Wunderlich

**Affiliations:** 1 Department of Internal Medicine II, University Hospital Würzburg, Würzburg, Germany; 2 Translational Medicine, Incyte Corporation, Wilmington, DE, United States; 3 Department of Pathology, Hematopathology Section and Lymph Node Registry, University Hospital Schleswig-Holstein, Kiel, Germany; 4 Department of Hematology, Odense University Hospital, Odense, Denmark; 5 Medizinische Klinik und Poliklinik II, Universitätsklinikum Würzburg, Würzburg, Germany; 6 Computational Drug Discovery, Incyte Corporation, Wilmington, DE, United States; 7 Institute of Pathology, University of Würzburg, Würzburg, Germany

**Keywords:** CAR-T, CD19, clinical trial, DLBCL, follicular lymphoma, immunotherapy, real-world, tafasitamab

## Abstract

**Introduction:**

The potential risk of eliminating or reducing CD19 expression following CD19-targeted therapies has raised questions about the optimal treatment sequencing strategies for B-cell malignancies. This study assessed CD19 expression and mutational status in samples from patients with relapsed or refractory B-cell malignancies who received tafasitamab, an anti-CD19 monoclonal antibody.

**Methods:**

Biopsy and peripheral blood samples were collected from a total of 100 patients with relapsed or refractory B-cell malignancies who received tafasitamab in a clinical trial or a real-world setting. Post-treatment samples collected from 66 patients were available for assessment of CD19 protein expression; six samples were excluded from the analyses as CD19 expression could not be assessed due to lack of residual lymphoma cells. The remaining 60 samples were assessed by either immunohistochemistry (n=46) or flow cytometry (n=14). Next-generation sequencing (NGS) was used to assess CD19 mutational status (n=39) and transcript expression (n=4). CD19 retention in lymphoma cell lines treated with tafasitamab was assessed by immunohistochemistry and flow cytometry.

**Results:**

Of 46 patient samples assessed by immunohistochemistry, 45 (98%) were CD19 positive after tafasitamab treatment. CD19 expression was retained in all 14 samples analyzed by flow cytometry. NGS of 39 samples revealed no somatic *CD19* mutations. CD19 expression was transiently masked in lymphoma cells treated with tafasitamab when assessed by flow cytometry; however, expression was detectable by immunohistochemistry at all time points assessed.

**Conclusion:**

These data suggest that tafasitamab treatment, even in the most recent line, may not preclude subsequent treatment with other CD19-targeting agents.

## 1 Introduction

The availability of CD19-targeting therapeutic options across a range of different drug classes, such as monoclonal antibodies (mAbs), antibody drug conjugates, and chimeric antigen receptor T-cell (CAR-T) therapies, allows for sequential targeting of CD19 in relapsed or refractory (R/R) B-cell lymphomas (1). The potential risk of eliminating or reducing CD19 expression following treatment with these agents has raised questions about the optimal treatment sequencing strategies, as treatment options for patients with CD19-negative disease at relapse could be limited (1). This concern is particularly relevant in diffuse large B-cell lymphoma (DLBCL), for which several CD19-directed therapies, including autologous CD19-targeted CAR-T therapies, are available for second-line treatment and beyond (2). CD19-negative relapse following CD19-directed CAR-T therapy, such as with axicabtagene ciloleucel, has been reported in approximately 30% of patients (3–5). Potential mechanisms leading to reduced CD19 availability following targeted therapy include loss of recognition domains due to somatic mutations or altered splicing, altered intracellular trafficking to the cell membrane, selective expansion of CD19-negative lymphoma cells, and epigenetic and transcriptional regulation (3, 6–12).

Tafasitamab is an anti-CD19 mAb with enhanced Fc function compared with the anti-CD20 mAb rituximab (13). Tafasitamab is a chemotherapy-free treatment approved in combination with lenalidomide for adults with R/R DLBCL who are ineligible for autologous stem cell transplant, and in combination with lenalidomide and rituximab for adults with R/R follicular lymphoma (FL) (13). Upon binding CD19, tafasitamab mediates antibody-dependent cellular cytotoxicity and antibody-dependent cellular phagocytosis, and exerts direct cytotoxicity (14, 15). A case study of longitudinal samples from 2 patients with CD19-positive non-Hodgkin lymphoma (NHL) suggested that treatment with tafasitamab resulted in transient CD19 masking (16). Conversely, several studies have reported retention of CD19 expression after tafasitamab treatment; however, the detection assays used were not standardized and sample sizes were limited (17–19). Determination of CD19 expression status following tafasitamab treatment may inform treatment decisions for patients with R/R B-cell malignancies. Herein, we report results from multiple complementary, standardized analyses that assessed CD19 expression and mutation status in post-treatment samples from patients who received tafasitamab as part of a clinical trial or in a real-world setting.

## 2 Methods

### 2.1 Patient samples

Post-treatment biopsy or peripheral blood samples were collected from a total of 100 patients with R/R B-cell malignancies who had no prior exposure to anti-CD19 agents (**Table 1**). Samples were collected from patients who received tafasitamab in the following studies: a phase 1 study of tafasitamab monotherapy in patients with chronic lymphocytic leukemia (CLL) (NCT01161511) (17); the phase 1/2 topMIND study in patients with NHL or CLL (NCT04809467); the identically designed phase 2 L-MIND (20) (NCT02399085) and phase 3 firmMIND (NCT05429268) studies in patients with R/R DLBCL (21) (**Supplementary Figure 1**); the phase 3, double-blind, two-arm, randomized, placebo-controlled inMIND study of tafasitamab in combination with lenalidomide and rituximab in patients with R/R FL or marginal zone lymphoma (NCT04680052) (22) (**Supplementary Figure 1**); and samples were also analyzed from patients with R/R DLBCL who received tafasitamab in a real-world setting according to the R/R DLBCL-approved regimen and dosing at the University Hospital Würzburg (**Table 1**). Patients in the real-world cohort were not transplant eligible, were mainly in second-line treatment, and did not receive tafasitamab as a bridging therapy. No cases of double or triple hit DLBCL were detected in the real-world cohort.

**Table 1:** Patient samples analyzed in the study.

Study name (ClinicalTrials.gov Identifier)	Treatment received	Disease	Assay (Samples, n)
Safety and tolerability of XmAb^®^5574 in CLL (NCT01161511)	Tafasitamab	R/R CLL	FC (14)
topMIND (NCT04809467)	Tafasitamab + parsaclisib	R/R NHL or CLL	NGS (7)
L-MIND (NCT02399085)	Tafasitamab + len	R/R DLBCL	IHC (6), NGS (4)
firmMIND (NCT05429268)	Tafasitamab + len	R/R DLBCL	IHC (10)^a^
inMIND (NCT04680052)	Tafasitamab + len + R	R/R FL or MZL	IHC (14)^b^ NGS (28)^c^
Real world (not applicable)	Tafasitamab + len^d^	R/R DLBCL	IHC (22)

^a^ One patient sample was CD19 negative at baseline and was excluded from IHC analyses. ^b^Five patient samples did not contain residual lymphoma cells and were excluded from IHC analyses. ^c^NGS was performed on samples from patients who were minimal residual disease positive after treatment. ^d^According to the R/R DLBCL-approved regimen and dosing.

CLL, chronic lymphocytic leukemia; DLBCL, diffuse large B-cell lymphoma; FC, flow cytometry; FL, follicular lymphoma; IHC, immunohistochemistry; len, lenalidomide; MZL, marginal zone lymphoma; NGS, next-generation sequencing; NHL, non-Hodgkin lymphoma; R, rituximab; R/R, relapsed/refractory.

### 2.2 Immunohistochemistry on patient samples

Immunohistochemistry (IHC) was performed on biopsy samples from patients in the studies indicated in **Table 1**. CD19 expression was determined based on the percentage of positive lymphoma cells and the average intensity score (1–3+) using clone LE-CD19 or BT51E. The sample was considered positive if staining was present on ≥10% of lymphoma cells. Six samples were excluded from the IHC analyses: one sample from firmMIND that was CD19 negative at baseline and five samples from inMIND that did not contain residual lymphoma cells after treatment (**Table 1**). The IHC assay was developed and extensively validated in a Clinical Laboratory Improvement Amendments/College of American Pathologists-certified laboratory using the anti-CD19 mAb LE-CD19 clone, which binds to an intracellular CD19 epitope and does not compete with tafasitamab. This mAb is commonly used in clinical practice to measure CD19 expression in patients. IHC on patient samples was also performed with CD19 mAb clones 109, CD19/3117, and ZR212, which were validated and established on tonsil and lymph node tissue using the Leica BOND system. All antibodies were cross-validated on a control block using the SU-DHL-4 DLBCL cell line and patient samples (data not shown and **Supplementary Figure 3**).

### 2.3 Next-generation sequencing

Next-generation sequencing (NGS) was performed with DNA and RNA extracted from patient samples. DNA samples from biopsies were mapped using the Illumina DRAGEN Bio-IT Platform 3.4 (Illumina, Inc., San Diego, CA, USA) and variants annotated using VarSeq software 2.2.1-SP3 (Golden Helix, Inc., Bozeman, MT, USA). *CD19* variants from unmatched DNA whole exome sequencing were assessed using Ingenuity^®^ Variant Analysis™ (Qiagen GmbH, Hilden, Germany). Peripheral blood DNA was sequenced using the validated Lymphoid 133 Panel (NeoGenomics Laboratories, Fort Myers, FL, USA). *CD19* sequencing of peripheral blood and bone marrow samples of minimal residual disease-positive patients from inMIND was performed using NovaSeq6000 (NeoGenomics).

### 2.4 Flow cytometry and IHC in preclinical models

SU-DHL-4 and Jurkat T lymphocytes (the latter used as a CD19-negative control) were either untreated or treated with tafasitamab 50 µg/mL for 24 hours in culture. After three washes, cells were cultured for 2, 5, or 24 hours before analysis. For flow cytometry, CD19 expression levels were analyzed with 1 µg/mL anti-CD19 clone HIB19 that competes with tafasitamab. For IHC, aliquots of the same SU-DHL-4 cells were fixed in formalin and embedded in paraffin and analyzed using anti-CD19 LE-CD19, 109, CD19/3117, and ZR212 mAb clones as above. The CD19-positive SU-DHL-2 cell line was used in flow cytometry assays only, using the experimental conditions described above.

## 3 Results

**Figure 1:**
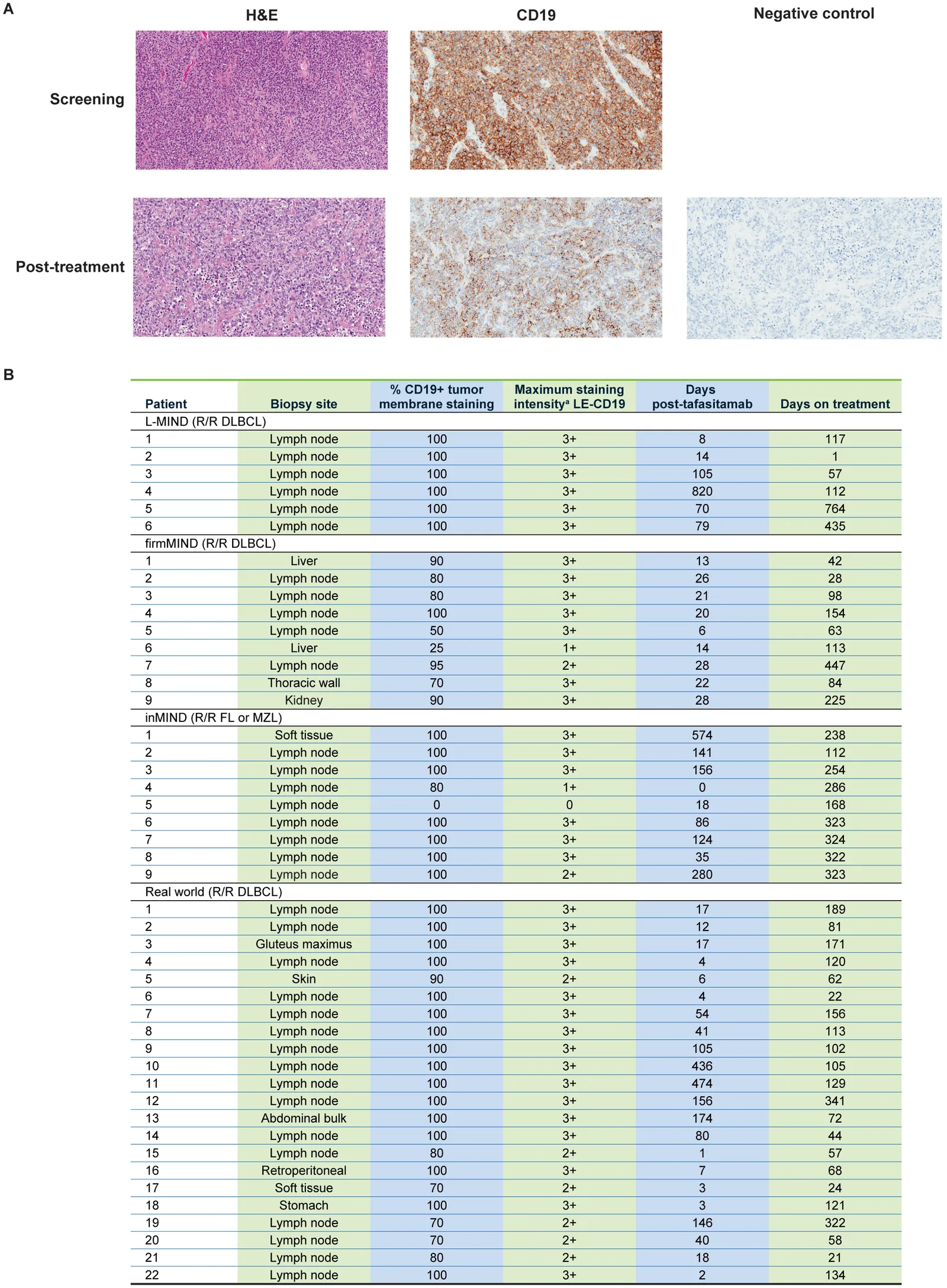
CD19 expression is retained in patient samples following tafasitamab treatment. **(A)** CD19 expression in representative patient samples from firmMIND at screening and after treatment with tafasitamab. H&E, negative control, and CD19 (clone LE-CD19) staining at 20× magnification is shown for patient 3. At screening, 95% of nucleated cells were lymphoma cells, which decreased to 55% after treatment. **(B)** CD19 expression by IHC after tafasitamab treatment in patients with R/R B-cell lymphomas enrolled in L-MIND (n=6), firmMIND (n=9), inMIND (n=9), and in real-world patients (n=22). ^a^Staining intensity: 0, negative; 1+, weak positive; 2+, moderate positive, 3+, strong positive. DLBCL, diffuse large B-cell lymphoma; FL, follicular lymphoma; H&E, hematoxylin and eosin; MZL, marginal zone lymphoma; R/R, relapsed/refractory.

Representative images demonstrating the maintenance of CD19 expression in biopsy samples from firmMIND following tafasitamab treatment are shown in **Figure 1A**. As of the data cutoff date (December 18, 2025), post-treatment samples from a total of 60 patients with R/R B-cell lymphomas were analyzed by either IHC or flow cytometry (**Table 1**). Of 46 patient samples analyzed by IHC, 45 (98%) were CD19 positive after tafasitamab treatment (**Figure 1B**). The post-treatment CD19-negative sample was from a patient with FL who was enrolled to the inMIND study and was refractory to rituximab. This patient’s baseline biopsy demonstrated weak staining for CD19 in 20% of lymphoma cells, yet they achieved a partial response. This finding may suggest CD19-positive cells were eliminated by treatment with tafasitamab, resulting in a partial response. It is also possible that the tafasitamab-based regimen resulted in the partial response followed by loss of CD19 expression. The observed partial loss of CD19 expression in the screening sample is unexpected for a patient with FL who had not received prior CD19-targeted therapy and suggests that clonal selection of CD19-negative cells prior to treatment could also have contributed to the partial response and residual CD19 loss.

Among the 46 samples assessed by IHC, the median duration from last tafasitamab dose to post-treatment biopsy collection was 27 days (range, 0–820). In the majority of samples, 100% of lymphoma cells expressed CD19 and staining intensity was scored as 3+ (strong positive). IHC summary data are presented in **Supplementary Figure 2**. These data are consistent with previously published results from a phase 1 study of tafasitamab in which 14 post-treatment samples from patients with CLL were assessed by flow cytometry and all stained positive for CD19 (17).

Among 22 patients who received tafasitamab in the real-world setting, three patients had sequential biopsies available. IHC analysis of these samples further demonstrated the robustness of CD19 expression following tafasitamab treatment and in the case of one patient, showed an increase in the proportion of CD19-positive lymphoma cells and staining intensity over time (**Supplementary Table 1**). Consistent with these observations, a greater proportion of samples collected at least three weeks from the last tafasitamab dose had 100% CD19-positive membrane staining on lymphoma cells and a maximum staining intensity of 3+, compared with those samples collected within three weeks of the last dose (**Supplementary Table 2**). Mean CD19-positive membrane staining intensity was also increased in samples collected at least three weeks from the last tafasitamab dose compared with samples collected within three weeks of the last dose.

Eight patients from the real-world cohort received subsequent CD19-directed CAR-T therapy with axicabtagene ciloleucel. The median duration between the last tafasitamab dose and the initiation of CAR-T therapy was 61 days (range, 7–77). Following CAR-T therapy, complete responses were recorded for four patients (50%); the remaining four patients experienced progressive disease. Although a small number of patients received CAR-T in this dataset, the complete response rate is consistent with overall CAR-T efficacy rate in real-world data (23). Our data are also in agreement with a recent study of 338 patients with R/R DLBCL who received CAR-T therapy following tafasitamab treatment, with an overall response rate of 81% and a complete response rate of 59% (24).

**Figure 2:**
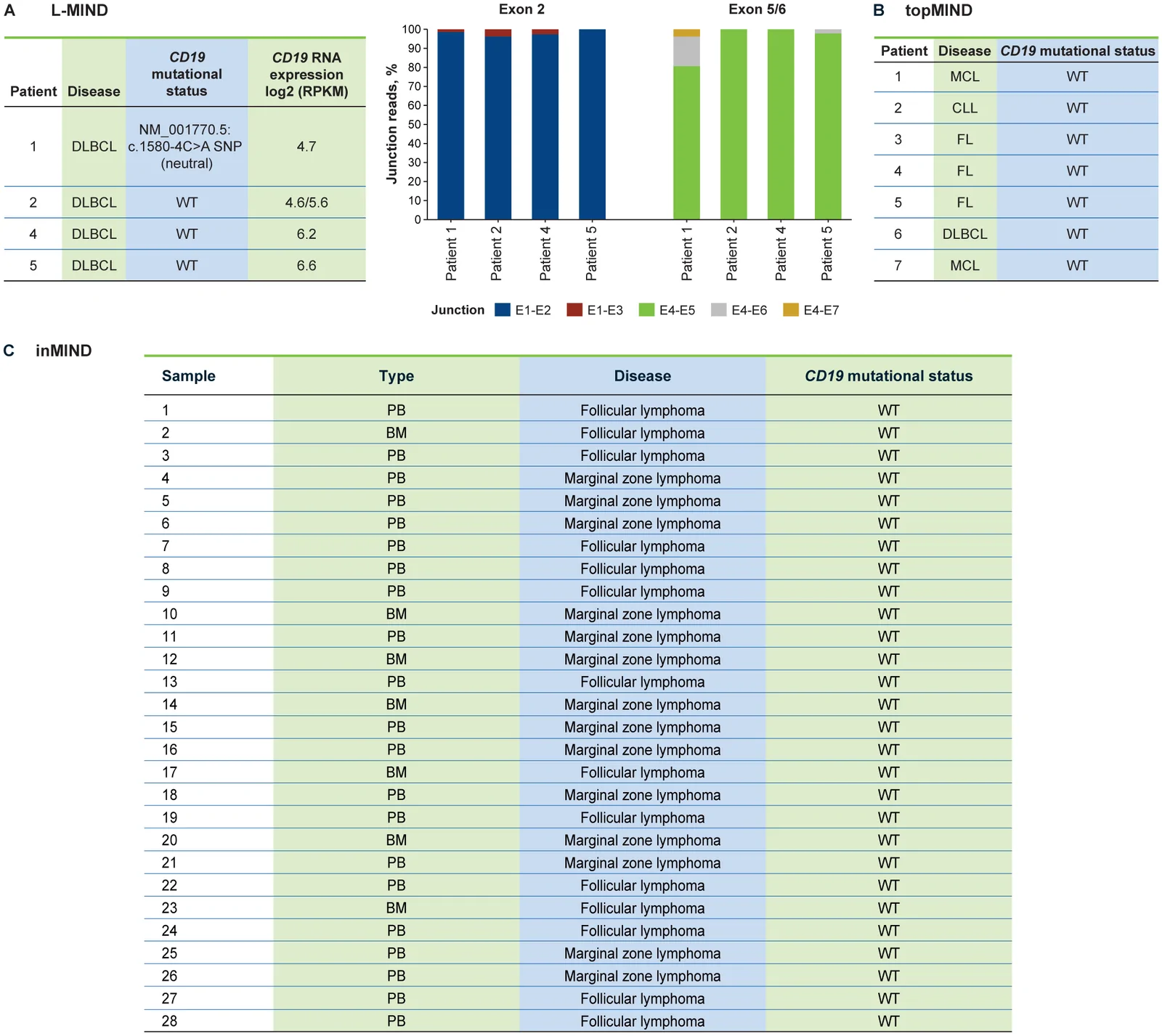
CD19 expression and mutational status assessed by next-generation sequencing. **(A)** *CD19* RNA expression was preserved in samples from L-MIND; minimal exon skipping was observed for exons 2 (E1 -E3), 5 (E4 -E6), and 5/6 (E4 -E7) in one patient. **(B, C)** No somatic *CD19* mutations were observed after tafasitamab treatment in samples from topMIND and inMIND. BM, bone marrow; CLL, chronic lymphocytic leukemia; DLBCL, diffuse large B-cell lymphoma; FL, follicular lymphoma; MCL, mantle cell lymphoma; PB, peripheral blood; RNA, ribonucleic acid; RPKM, reads per kilobase of transcript per million mapped reads; SNP, single nucleotide polymorphism; WT, wild-type.

NGS analysis of 39 patient samples from three clinical trials revealed no somatic *CD19* mutations after tafasitamab treatment (**Figure 2**). However, four common population variants were found, and all seven blood samples from topMIND displayed alterations in other genes commonly mutated in lymphoma. *CD19* expression was preserved in all four samples from L-MIND that were RNA sequenced; minimal exon skipping was observed for exons 2 (E1–E3), 5 (E4–E6), and 5/6 (E4–E7) in one patient (**Figure 2**). In total, samples from 5 patients were analyzed by both NGS and IHC (L-MIND patients 1, 2, 4, and 5, and inMIND patient 8). In this subgroup of samples, CD19 was expressed on 100% of lymphoma cells with a maximum staining intensity of 3+, indicating robust expression of wild-type CD19 after tafasitamab treatment (**Figure 1B**).

To further evaluate the suitability of commercially available anti-CD19 mAbs targeting both intra- and extracellular epitopes for CD19 IHC analysis after tafasitamab treatment, we cross-validated a panel of three other mAbs that are commonly used in clinic on control cell line blocks (not shown) and patient biopsy samples. Notably, all tested mAbs detected CD19 staining post treatment, despite variable durations between the time of the last tafasitamab dose and biopsy collection (**Supplementary Figure 3**).

**Figure 3:**
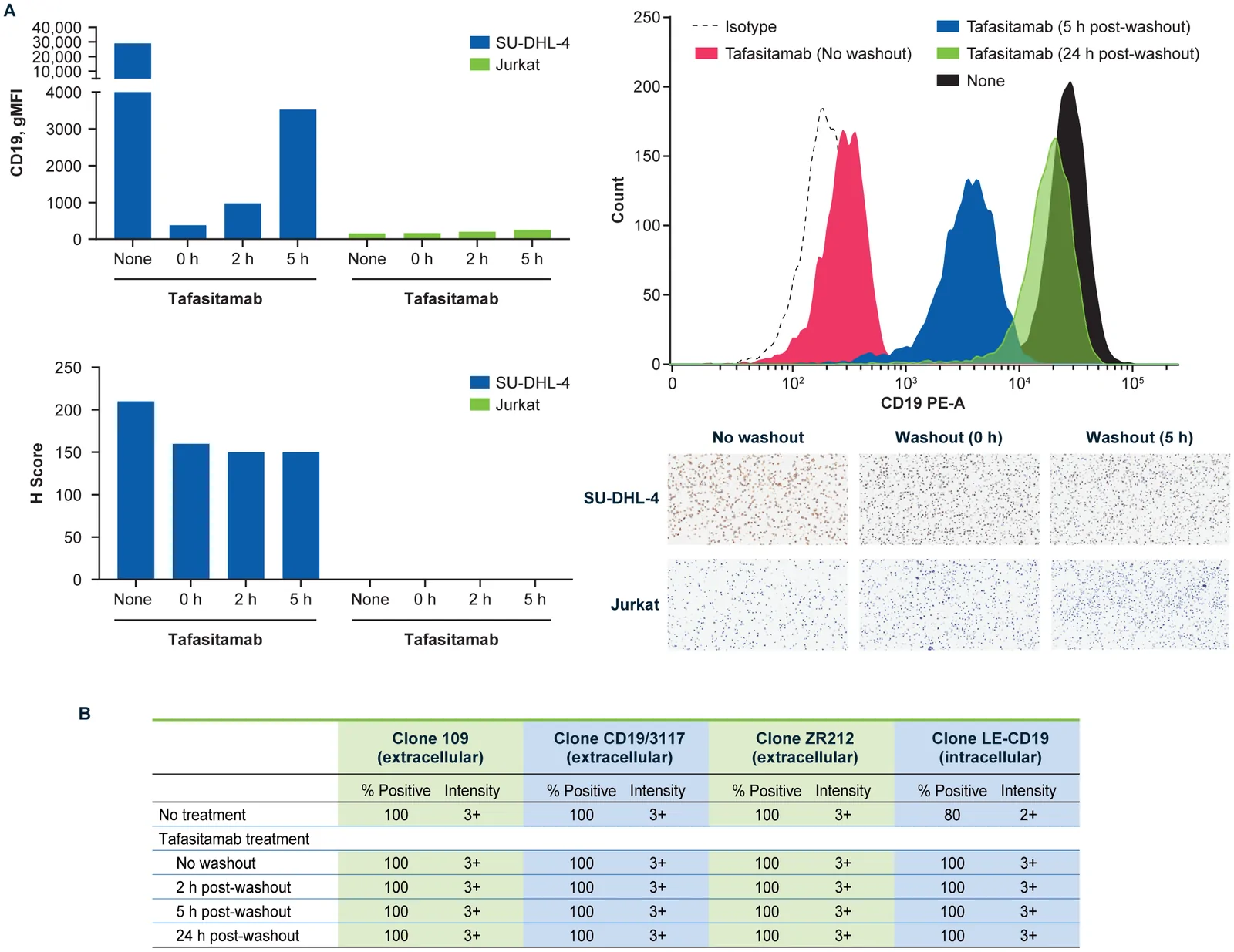
CD19 expression after tafasitamab treatment in preclinical models. **(A)** CD19 expression was masked after tafasitamab treatment when assessed by flow cytometry but detectable by IHC. After tafasitamab treatment and followed by a wash-out, CD19 expression gradually increased to a nearly untreated level in 24 hours. Representative histogram (top right; no washout [red], 5 hours after washout [blue]), and 24 hours after washout [green]). Geometric mean fluorescence intensities (top left) over a 5-hour course of tafasitamab treatment showed a transient increase in CD19 expression in SU-DHL-4 cells compared with the Jurkat negative control. Using IHC, CD19 expression was detected on SU-DHL-4 cells independent of tafasitamab treatment at all time points evaluated, but not on CD19 negative control Jurkat cells. Representative images are shown on the bottom right, and quantification is on bottom left. Data are representative of two independent experiments. **(B)** IHC analysis of the SU-DHL-4 blocks generated from cells in **(A)** with or without tafasitamab washout showed similar trends using a series of anti-CD19 mAbs with either intracellular or extracellular epitopes. gMFI, geometric mean fluorescence intensity; IHC, immunohistochemistry; mAb, monoclonal antibody; PE, phycoerythrin.

Consistent with the results of a previous study (25), the addition of tafasitamab to CD19-positive SU-DHL-4 lymphoma cells resulted in CD19 masking, rendering the receptor undetectable by flow cytometry (**Figure 3A**). This effect was reversed upon tafasitamab washout from culture media, which resulted in CD19 expression returning to baseline levels approximately 24 hours after tafasitamab removal. Transient masking of CD19 by tafasitamab was confirmed in flow cytometry experiments using SU-DHL-2 lymphoma cells, which express CD19 at a low level (**Supplementary Figure 4**). In contrast to data obtained by flow cytometry, CD19 was detectable by IHC on nearly all SU-DHL-4 lymphoma cells at all time points tested using a range of anti-CD19 mAbs, which was consistent with data obtained in patient samples (**Figure 3B**; **Supplementary Figure 3**).

## 4 Discussion

To date, using standardized clinically validated assays, we have observed retention of the CD19 antigen in 59/60 (98%) samples with residual lymphoma cells from patients with R/R B-cell malignancies after receiving tafasitamab; no somatic *CD19* mutations were observed. All intra- and extracellular anti-CD19 antibodies tested were considered suitable for assessing CD19 expression by IHC after tafasitamab treatment. Importantly, CD19 expression was retained independent of B-cell malignancy and in patient samples collected across a wide range of durations from the last tafasitamab dose, including ten samples collected within seven days of the last dose. CD19-positive samples collected during tafasitamab treatment (inMIND patient 4) or 1-day post - treatment (real-world patient 15) support that tafasitamab does not cause loss of CD19 expression in the short term. Notably, 76% of post-treatment IHC-determined intensity scores were 3+, indicating robust CD19 expression. These complementary analyses of samples from patients with a range of R/R B-cell malignancies support and expand upon data from previous studies that demonstrated retention of CD19 expression following tafasitamab treatment (17–19). Although limited by sample size, the observed 50% complete response rate in patients with DLBCL who received axicabtagene ciloleucel following tafasitamab in this study is consistent with real-world response rates for CAR-T therapy and these data are supportive of previous studies demonstrating the efficacy of CAR-T therapy after treatment with tafasitamab (23, 24, 26, 27).

In conclusion, the results of these analyses demonstrate that CD19 expression was retained in samples from patients with R/R B-cell malignancies who received tafasitamab in either clinical trial or real-world settings. Importantly, CD19 expression was observed in patient samples collected 0–820 days post treatment, suggesting that treatment with tafasitamab did not result in CD19 loss. Furthermore, no somatic *CD19* mutations were detected in samples from patients who received tafasitamab in clinical trials. Together, these data suggest that previous tafasitamab exposure, even in the most recent line, should not preclude subsequent treatment with CD19-targeting agents, such as CAR-T therapy (24, 26, 27). Additional studies with increased sample size are ongoing to extend these findings.

## Data Availability

The datasets presented in this article are not readily available due to patient confidentiality; nevertheless, Incyte Corporation (Wilmington, DE, USA) is committed to data sharing that advances science and medicine while protecting patient privacy. Qualified external scientific researchers may request anonymized datasets owned by Incyte for the purpose of conducting legitimate scientific research. Researchers may request anonymized datasets from any interventional study (except phase I studies) for which the product and indication have been approved on or after January 1, 2020, in at least 1 major market (e.g., US, EU, JPN). Data will be available for request after the primary publication or 2 years after the study has ended. Information on Incyte’s clinical trial data sharing policy and instructions for submitting clinical trial data requests are available at: https://www.incyte.com/Portals/0/Assets/Compliance%20and%20Transparency/clinical-trial-datasharing.pdf?ver=2020-05-21-132838-960.
