# Supplementary material for: CD19 expression is preserved following CD19-directed monoclonal antibody therapy with tafasitamab
Source: Front Immunol. 2026 Sep 3;17:1878229. doi: 10.3389/fimmu.2026.1878229 (PMC13583598; doi:10.3389/fimmu.2026.1878229)
Supplement: Supplementary file 1 [file DataSheet1.pdf]

*Supplementary Material*

Supplementary Table 1. CD19 expression in serial biopsies taken from three patients who received tafasitamab in the real-world setting.

| Patient | Biopsy site | CD19-positive membrane staining, % | Maximum staining intensity[a] (zytomed) | Days post tafasitamab | Days on treatment |
|---|---|---|---|---|---|
| **Real world (R/R DLBCL)** | | | | | |
| 3a | Gluteus maximus | 100 | 3+ | 17 | 171 |
| 3b | Gluteus maximus | 100 | 3+ | 21 | |
| 5a | Skin | 90 | 2+ | 6 | 62 |
| 5b | Lymph node | 100 | 3+ | 105 | |
| 6a | Lymph node | 100 | 3+ | 4 | 22 |
| 6b | Preauricular | 100 | 3+ | 76 | |

Serial biopsies were collected at different times after tafasitamab treatment from three patients, demonstrating the robustness of CD19 expression. [a]Staining intensity: 0, negative; 1+, weak positive; 2+, moderate positive, 3+, strong positive. DLBCL, diffuse large B-cell lymphoma; R/R, relapsed/refractory.

Supplementary Table 2. CD19 expression in biopsies grouped by time post tafasitamab dose.

| **Variable** | **Biopsy ≤ 21 days post tafasitamab (n=21)** | **Biopsy > 21 days post tafasitamab (n=25)** |
|---|---|---|
| 100% CD19-positive membrane staining, n (%) | 11 (52.4) | 19 (76.0) |
| Mean CD19-positive membrane staining intensity, % | 83.1 | 95.0 |
| 3+ maximum staining intensity, n (%) | 14 (66.7) | 21 (84.0) |

The proportion of samples with 100% CD19-positive membrane staining, mean CD19-positive membrane staining intensity, and proportion of samples with 3+ staining intensity was increased in samples collected >21 days post tafasitamab compared with those collected ≤21 days post tafasitamab.



This is a provisional file, not the final typeset article

Supplementary Figure 1. L-MIND, firmMIND, and inMIND study design and biopsy collection. [a]Tafasitamab 12 mg/kg IV or placebo (0.9% saline solution IV) was administered Q4W during cycles 1 to 3 (days 1, 8, 15, 22), and Q2W during cycles 4 to 12 (days 1 and 15) and thereafter. Treatment with tafasitamab could continue until disease progression or unacceptable toxicity. In L-MIND and firmMIND, a loading dose of tafasitamab was administered at cycle 1, day 4. [b]Lenalidomide 20 mg PO QD was administered on days 1 to 21 of cycles 1 to 12 at approximately the same time every day. [c]Rituximab 375 mg/$m^2$ IV was administered Q4W during cycle 1 (days 1, 8, 15, 22) and Q1W during cycles 2 to 5 (day 1). ASCT, autologous stem cell transplant; C1D1, cycle 1 day 1; DLCBL, diffuse large B-cell lymphoma; ECOG PS, Eastern Cooperative Oncology Group performance status; EOT, end of treatment; FFPE, formalin-fixed paraffin-embedded; FL, follicular lymphoma; HDC, high-dose chemotherapy; IV, intravenous; MZL, marginal zone lymphoma; PO, orally; Q1W, every week; Q2W, every 2 weeks; Q4W, every 4 weeks; QD, once daily; R/R, relapsed or refractory.

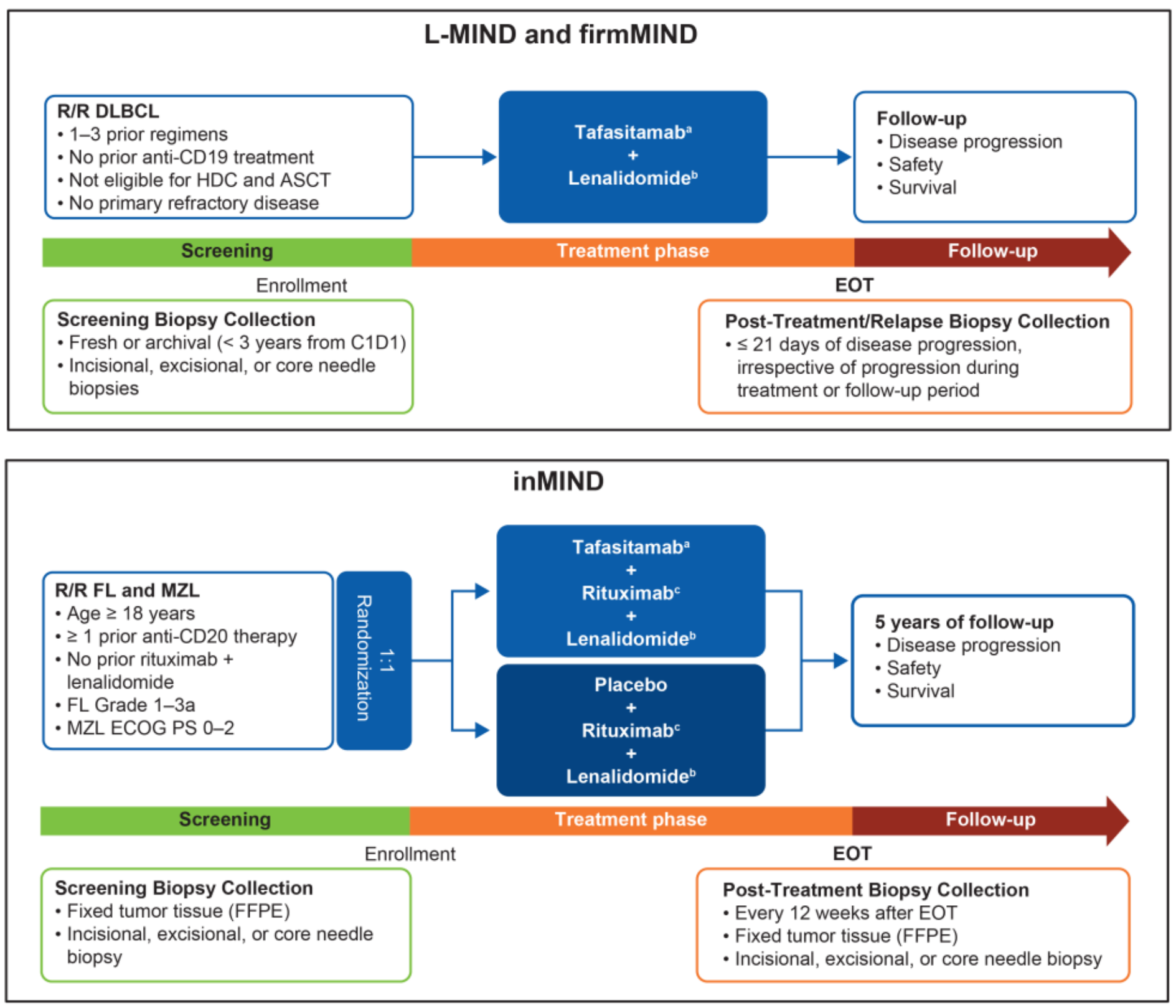

Supplementary Figure 2**.** Quantification of CD19 expression and staining intensity in samples analyzed by immunohistochemistry. Quantification of CD19 membrane staining positivity and staining intensity scores for the 46 samples included in Figure 1 are presented. Data above each bar report the percentage of samples within the respective categories. [a]Staining intensity: 0, negative; 1+, weak positive; 2+, moderate positive, 3+, strong positive.

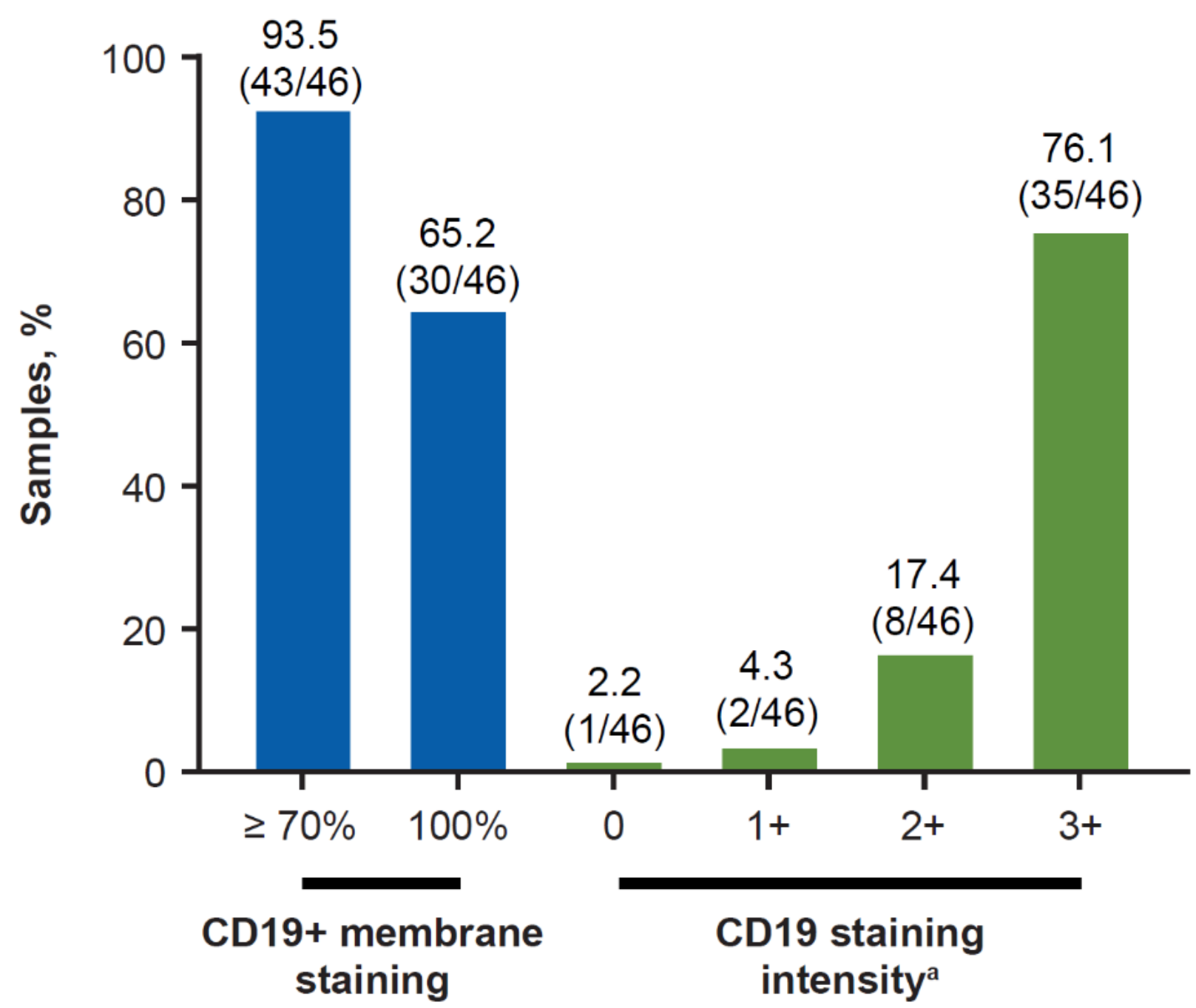




This is a provisional file, not the final typeset article

Supplementary Figure 3. CD19 expression assessed with a panel of antibodies in patient samples following tafasitamab treatment. CD19 expression was assessed using commercially available monoclonal anti-CD19 antibodies. All images are presented at 5x magnification, except for patient 1 post-treatment images, which are at 20x magnification. All slides were scanned with PANNORAMIC SCAN II BF (3DHISTECH).

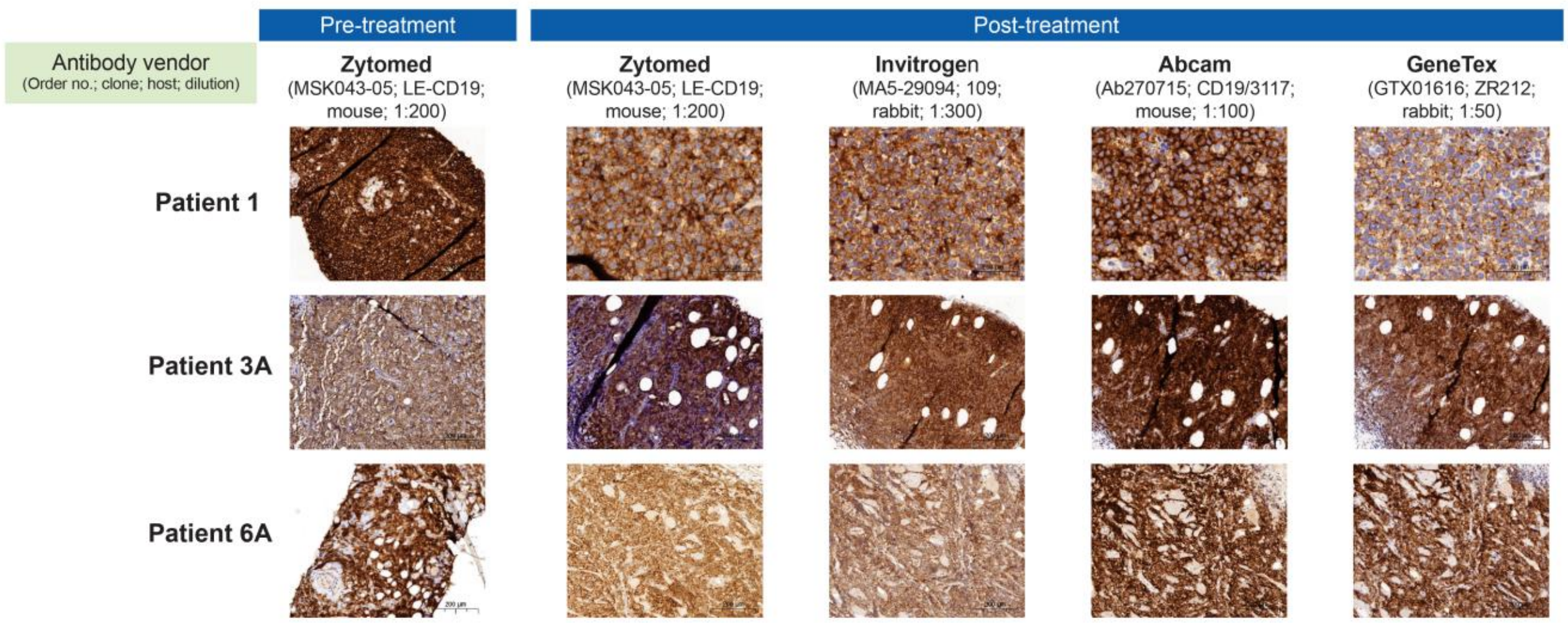

Supplementary Figure 4. CD19 expression in SU-DHL-2 cells assessed by flow cytometry. SU-DHL-2 cells. Representative histograms demonstrating CD19 expression under normal culture conditions (black), in the presence of tafasitamab (red), 5 hours after tafasitamab washout (blue), and 24 hours after tafasitamab washout (green).

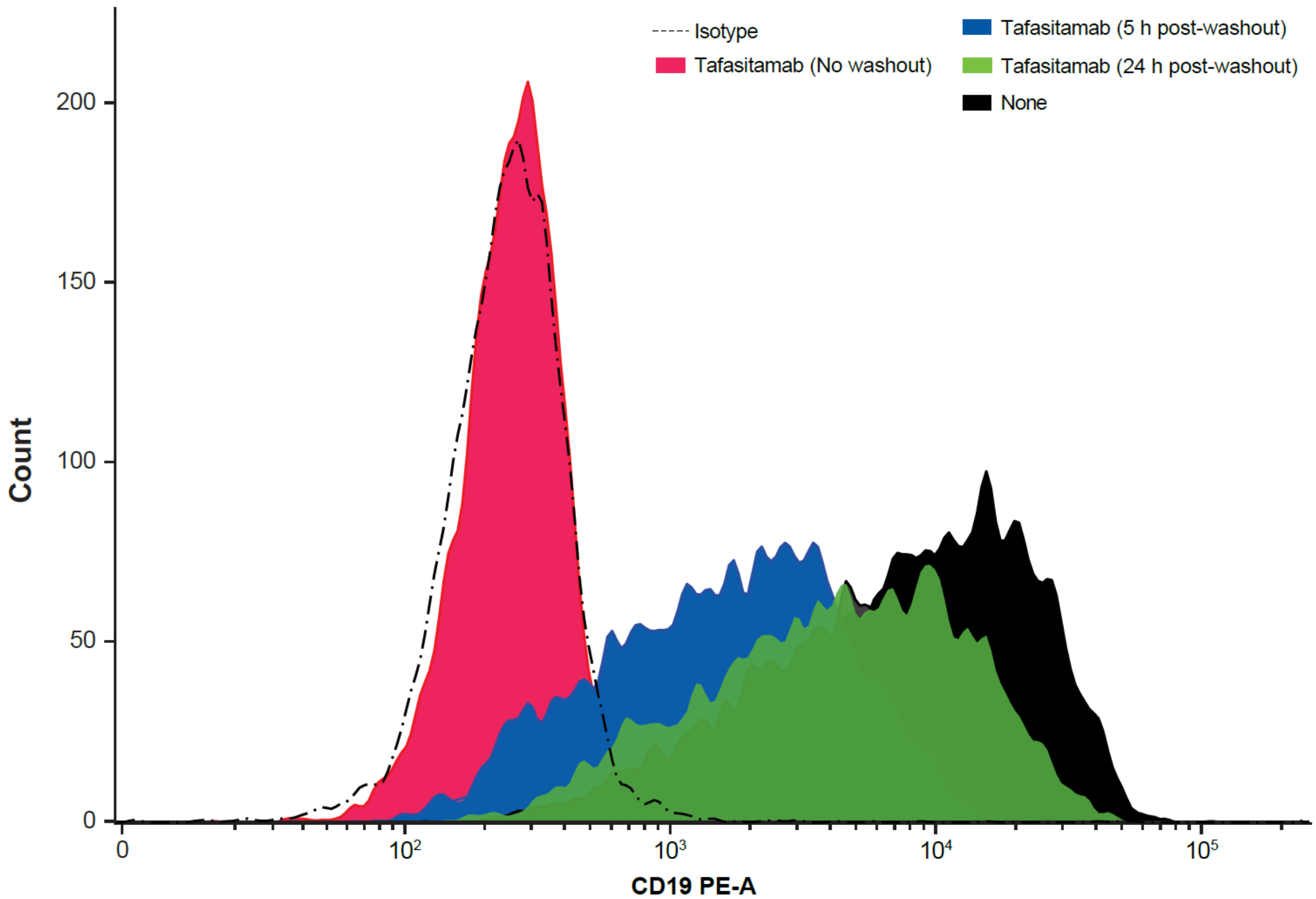




This is a provisional file, not the final typeset article
